# Dual Reward Prediction Components Yield Pavlovian Sign- and Goal-Tracking

**DOI:** 10.1371/journal.pone.0108142

**Published:** 2014-10-13

**Authors:** Sivaramakrishnan Kaveri, Hiroyuki Nakahara

**Affiliations:** 1 Lab for Integrated Theoretical Neuroscience, RIKEN BSI, Wako, Japan; 2 Dept. of Computational Intelligence and Systems Science, Tokyo Institute of Technology, Yokohama, Japan; Duke University Medical Center, United States of America

## Abstract

Reinforcement learning (RL) has become a dominant paradigm for understanding animal behaviors and neural correlates of decision-making, in part because of its ability to explain Pavlovian conditioned behaviors and the role of midbrain dopamine activity as reward prediction error (RPE). However, recent experimental findings indicate that dopamine activity, contrary to the RL hypothesis, may not signal RPE and differs based on the type of Pavlovian response (e.g. sign- and goal-tracking responses). In this study, we address this discrepancy by introducing a new neural correlate for learning reward predictions; the correlate is called “cue-evoked reward”. It refers to a recall of reward evoked by the cue that is learned through simple cue-reward associations. We introduce a temporal difference learning model, in which neural correlates of the cue itself and cue-evoked reward underlie learning of reward predictions. The animal's reward prediction supported by these two correlates is divided into sign and goal components respectively. We relate the sign and goal components to approach responses towards the cue (i.e. sign-tracking) and the food-tray (i.e. goal-tracking) respectively. We found a number of correspondences between simulated models and the experimental findings (i.e. behavior and neural responses). First, the development of modeled responses is consistent with those observed in the experimental task. Second, the model's RPEs were similar to dopamine activity in respective response groups. Finally, goal-tracking, but not sign-tracking, responses rapidly emerged when RPE was restored in the simulated models, similar to experiments with recovery from dopamine-antagonist. These results suggest two complementary neural correlates, corresponding to the cue and its evoked reward, form the basis for learning reward predictions in the sign- and goal-tracking rats.

## Introduction

Pavlovian conditioning is a major paradigm for studying associative learning between cues and rewards. Partly inspired by Pavlovian conditioning behavior, reinforcement learning (RL) and temporal difference (TD) learning in particular has become a dominant computational paradigm for understanding reward-based learning and decision-making as well as their underlying neural correlates [1]–[7]. A celebrated RL account posits that the phasic activity of midbrain dopamine (DA) neurons signals reward prediction error (RPE), the difference between expected and actual reward; the phasic DA activity is involved in learning reward predictions and associated behaviors [8], [9]. In the RL description of Pavlovian conditioning, the strength of Pavlovian responses evoked by a cue is proportional to the predicted reward, while phasic DA activity signals the response-independent difference between actual and predicted reward [10], [11].

**Figure 1:**
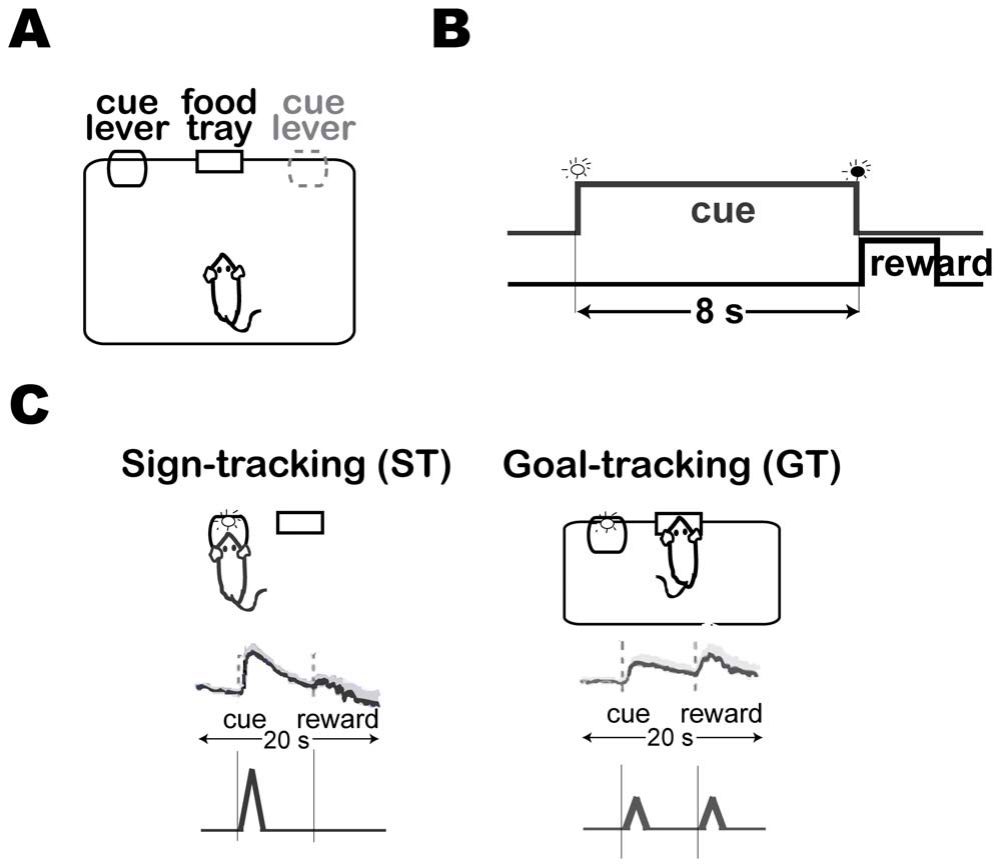
Schematic of the Pavlovian conditioned approach (PCA) task. (A) The apparatus. (B) Temporal order of events in a trial. (C) Illustrations of sign- and goal-tracking responses in the task (*top*) and respective DA responses (*middle, bottom*). (A) One of the two cues (illuminated-levers) is randomly assigned to each animal and then consistently used for all of the sessions. (B) In each trial, the cue was presented for 8s and then immediately followed by reward (food pellets in the food tray). (C) (*Top*) Two types of conditioned-approach behaviors are observed immediately during cue presentation; one group of rats approach the cue and stay at the cue until it is retracted at which time they approach the food-tray (*sign-tracking*); the other group approach the food-tray and wait for the reward (*goal-tracking*). (*Middle*) Phasic dopamine (DA) release recorded in the core of the nucleus accumbens using fast-scan cyclic voltammetry during cue and reward presentation in the final conditioning session [25]. (Bottom) Illustration of the phasic DA activity assumed in this study, based on the DA release recorded in the nucleus accumbens.

However, the RL account was recently challenged [12], [13] using a variant of Pavlovian conditioned approach (PCA) task [14], [15], wherein the reward (food pellets) was delivered in a food tray at a different location from the cue (illuminated-lever) (Figure 1A). The task was thus able to dissociate responses towards the cue from those towards the reward. Following paired presentations of the cue and the reward (Figure 1B), the rats in the study approached distinct locations during the cue presentation phase (Figure 1C) - some rats approached the cue (i.e. sign-tracking) while others approached the food-tray (i.e. goal-tracking) [14], [16], [17]. The two dissociable approach responses develop at the same rate in the corresponding groups of rats; it could be interpreted that both groups learned reward prediction at the same rate. The DA activity, measured as peak DA concentration in the core of nucleus accumbens (Nacc), was found to be different between the two groups: DA activity in the sign-tracking rats exhibited a large phasic response at the time of the cue and no response at the time of the reward (Figure 1C) while DA activity in the goal-tracking rats showed a relatively weaker phasic response at both the time of the cue and the reward (Figure 1C) [12]. The observed DA concentration challenges the RL account of DA activity: similar DA activity for similar reward predictions. Furthermore, the goal-tracking, but not sign-tracking, rats could learn cue-reward predictions under the influence of dopamine antagonist, i.e. in absence of dopamine RPE signals [12].

This study addresses the apparent discrepancy of these experimental findings with the RL description by clarifying the neural correlates underlying the reward prediction [18], [19]. Early conditioning theories, like Konorski's [20], and other recent works [21]–[24] have proposed that different properties of reward (e.g. location, reward type) may be transferred to the reward-predicting cue. These transferred properties of reward underlie different characteristic Pavlovian responses, i.e. observed variations in the experiments [22]. This notion of transferring reward properties is not well incorporated in the RL description. In this description, at the cue only a quantitative expectation of the reward is learned and associated response is induced. However, this description ignores the possibility that cue can evoke recall of rewards that in addition to the cue can participate in learning the reward predictions. The resulting reward prediction contains multiple components that can differentially contribute to behavior in conditioning tasks producing response variations observed in experiments like sign-tracking and goal-tracking.

**Figure 2:**
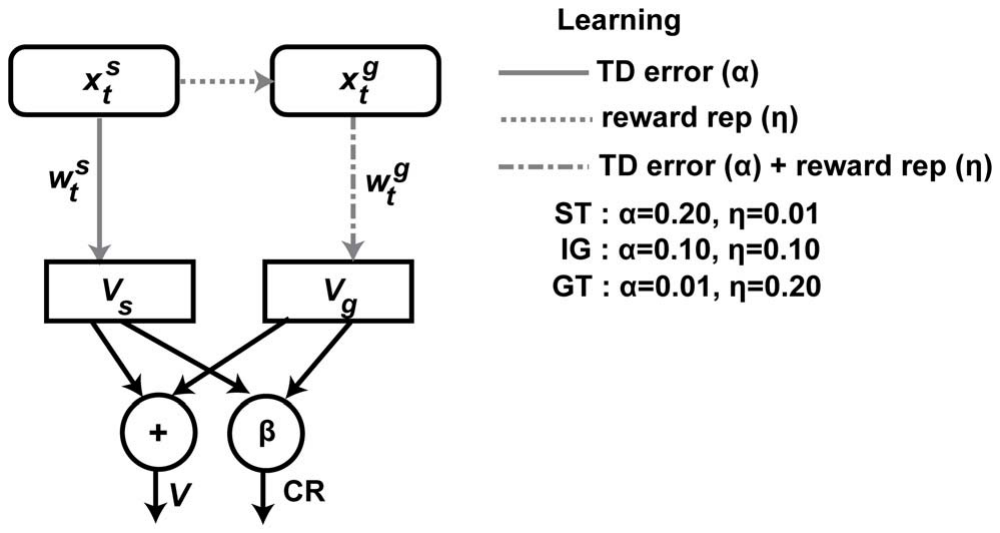
Schematic diagram of reward prediction and Pavlovian responses. Reward predictions (*V*(*t*)) are learned as the sum of the sign (*V_s_*(*t*)) and the goal component (*V_g_*(*t*)), which are based on neural correlates of cue (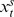) and cue-evoked recall of reward (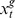) respectively. Upon the cue presentation, in addition to the standard cue correlate, the correlate of cue-evoked reward is generated (indicated by the dashed arrow from 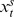 to 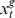). The TD error is used for learning both *V_s_*(*t*) and *V_g_*(*t*) (“TD Error”). On the other hand, the correlate of cue-evoked reward presentation is learned independent of TD error (“reward rep”), which further influences the learning of goal component (“TD error + reward rep”). Each reward prediction component *V_s_*(*t*) and *V_g_*(*t*) supports specific pavlovian responses directed towards the cue and the food tray respectively.

We used a simplified TD model that includes transfer of reward properties to the cue to study these intuitions on response variability (Figure 2). In this model, the reward prediction is learned, using the RPE, over two neural correlates – a correlate of the cue and a correlate of cue-evoked reward. We refer to the reward predictions assigned to the two neural correlates as sign and goal components respectively. The development of goal component may differ depending upon the rate at which the correlate of cue-evoked reward is learned. The sign and goal reward prediction components together contribute to the animal's net reward prediction and their relative magnitude determines the prevalence of sign- and goal-tracking in the PCA task. We suggest that these processes underlie the differential tendency for sign- and goal-tracking observed in the experiments. We simulated the model in the experiment, with differential learning of the two components, and observed a number of correspondences between the models and the different response groups in the experiment.

## Methods

We first describe the standard TD formulation applied to reward prediction and decision-making [2], [8], [10] and then provide description of our model. Reward prediction given a stimulus at time *t*, denoted by *V*(*t*), is computed based on neural correlates evoked by the stimulus denoted by *x_t_* (referred to as input). The reward prediction is given by
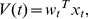
(1)where *w_t_* is the weight vector. The predictions are learned iteratively using learning signals referred to as temporal difference (TD) errors. On transition to the next time period (i.e., *t*+1), the TD error (*δ*(*t*)) is computed as: 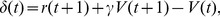
(2)where *r*(*t*) is the obtained reward (or its absence) at time *t* and *γ* is the discount factor ([0, 1]). Phasic DA activity is considered to encode this TD error signal. The reward prediction *V*(*t*) is updated using TD error, i.e. the weight vector is updated as follows:
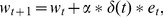
(3)where *α* is the learning rate ([0, 1]) and *e_t_* is the eligibility of the previous inputs. The eligibility trace is recursively computed as 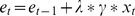 (with the initial value being *e*_0_ = 0) where *λ* is the decay parameter ([0, 1]) that determines the extent of updates due to TD error at time *t* (*δ*(*t*)): for instance, when *λ* = 0 only the weights of input at time t are eligible for update and when *λ* = 1 the weights of all previous inputs in the trial are eligible for update.

We built a simplified model that includes learning the correlate of cue-evoked reward in the standard TD formulation (Figure 2). We replaced the original input (Equation 1) with an input with two parts: the first part (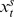) is the original input i.e. the neural correlate of the cue and the second part (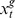) is the neural correlate of cue-evoked reward 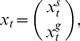
(4)


The correlate of cue-evoked reward is initialized to zeros at the start of the task, as no cue-reward pairing has been experienced yet, but is learnt by recursively updating, based on presence or absence of reward in the trial, to decrease the difference between 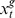 and observed reward, 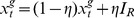
(5)where *I_R_* is 1 if reward is delivered in the trial, 0 otherwise. Thus the correlate of cue-evoked reward reflects an approximation of any rewards previously associated with the cue in the task. Correspondingly, the weight vector was also divided into two parts, i.e. 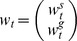. It is updated using standard TD error (Equations 2, 3).

The reward prediction (*V*(*t*)) and the correlate of cue-evoked reward (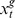) both contain estimates of expected reward in the trial. The reward prediction (*V*(*t*)) being a quantitative description of the prediction while the correlate of cue-evoked reward (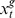) is a direct recall of rewards associated with the cue. We introduced another learning rule for 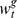 to take advantage of direct recall of rewards available in the correlate of cue-evoked reward (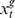). The correlate of cue-evoked reward (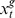) is used to update reward predictions, within the trial, independent of TD learning. 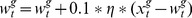
(6)


In our proposed model, the reward prediction can be learned through two distinct learning mechanisms – standard TD error dependent learning and learning through the correlate of cue-evoked reward that is independent of TD error. The TD error independent learning includes two processes – the learning of the correlate of cue-evoked reward (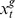; Equation 5) and the weight update based on 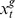 (Equation 6).

The reward prediction (*V*(*t*)) can be defined as sum of two components, 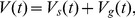
(7)each of which is given by 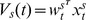 and 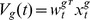 respectively.

We refer to *V_s_* and *V_g_* during the cue period, as the sign and goal components respectively. We associated these components to promote sign- and goal-tracking respectively. The relative magnitude of the components determines the probabilistic choice of sign- and goal-tracking responses during the cue period; the probability of sign-tracking is given by
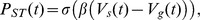
where σ(.) is the sigmoid function (
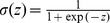
) and *β* is the so-called inverse temperature parameter that determines the influence of the relative magnitudes of the reward prediction components on the probability. The probability of goal-tracking is given by *P_GT_*(*t*) = 1−*P_ST_*(*t*).

In the model, we hypothesized that an animal's preference for sign- or goal-tracking in the PCA task depends on differential learning of sign and goal components. We instantiated this view by using different learning rates in the two distinct learning processes i.e. we used different learning rates, *α* and *η*, to model the responses of sign- and goal-tracking rats respectively: we used the set (*α*, *η*) = (0.2, 0.01) for sign-tracking group and (*α*, *η*) = (0.01, 0.2) for goal-tracking group. Otherwise all the parameters of the models were equal between the two groups and these were set to *γ* = 0.9, *λ* = 0.9 and *β* = 5.

We also extended our model to action-dependent reward predictions to study the models in SR task. In the standard RL account, action-dependent reward predictions for taking action *a* at time *t*, *Q_a_*(*t*), are defined as 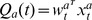, where 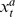 is the action specific input and 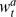 is the action-specific weight vector. The probability of choosing action *a* among two options (*a* and *b*) at time *t* is given by softmax function *P_a_*(*t*) = *σ*(*β*(*Q_a_*(*t*)−*Q_b_*(*t*))). On choosing action *a*, the action-dependent prediction error is computed as *δ_a_*(*t*) = *r*(*t*+1)+*γV*(*t*+1)−*Q_a_*(*t*) and is used to update *Q_a_*(*t*) i.e. update the weight vector 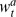, 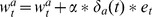, where *α* is the learning rate ([0, 1]) and *e_t_* is the eligibility of the previous inputs (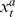). In the revised model of action-dependent reward predictions, we used an input with two parts 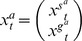: the first part (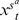) is the original action specific input and the second part (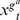) is the neural correlate of action-associated rewards (i.e. recall of rewards that follow the action). The corresponding weight vector was also divided into two parts, 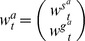. The revised model was used in the simulation of SR task with the parameters of the model set to be the same as in the PCA task.

In this study, we simulated the model in two experimental tasks [25]: a Pavlovian conditioned approach (PCA) task and a secondary reinforcement (SR) task, which measure different facets of animals' reward prediction learning.

### PCA task

The PCA task is used to access learning of cue-reward association by measuring the strength and direction of conditioned responses evoked by the cues [14], [26]. In this task, a cue and a reward are presented, either separately or together, repeatedly in random schedule. The rewards are delivered independently of the animals' responses to the cue. In Flagel et al [12], a retractable illuminated lever (cue) was presented, by inserting it into the chamber 2.5 cm to the left or right of the centrally located food tray, for 8 seconds. When the lever was retracted, one food pellet (reward) was delivered into the food-tray (Figure 1B: paired-condition). In each session (one session per day), the cue-reward pair was presented in 25 trials on a random interval 90-second schedule. A random condition was employed, in which the cue and reward were presented pseudo-randomly (the reward never occurred within 5 seconds of the cue). Each of these sessions also contained 25 presentations of the cue and reward. In a third condition (DA-blockade), a non-specific DA receptor antagonist (cis-flupenthixol) was systemically administered during “training” sessions (1–7) of the paired-condition; behavior was also measured in the 8th session when the DA receptor antagonist was not administered.

#### Simulation procedure

**Figure 3:**
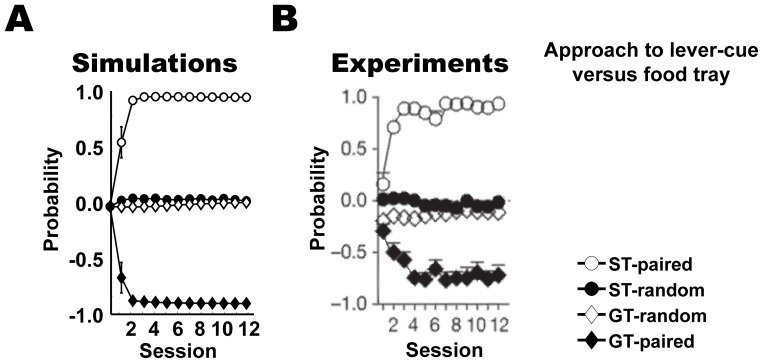
Pavlovian approach responses in the PCA task. Results showing the probability of approach responses, mean + s.e.m., of (A) the simulated models and (B) the animal experiments [25]. ST: sign-tracking, GT: goal-tracking. The plots show the probability of sign-tracking responses relative to the probability of goal-tracking responses in the range [−1, 1].

**Figure 4:**
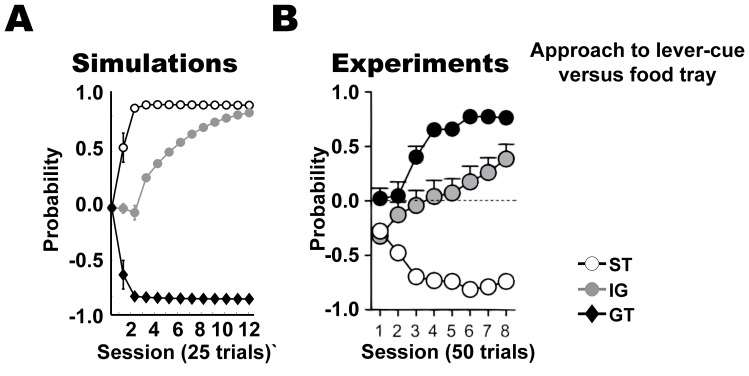
Responses of intermediate group (IG) in the PCA task. Results showing the probability of approach responses, mean + s.e.m., of (A) the simulated models and (B) the animal experiments [14]. ST: sign-tracking, GT: goal-tracking, IG: intermediate-group. The plot shows the probability of sign-tracking responses relative to the probability of goal-tracking responses in the range [−1, 1].

We simulated the three task conditions (paired, random, and DA-blockade) in the PCA task, using our TD model in an episodic (trial-by-trial) setting. Each trial comprised 100 discrete time bins (each time bin  = 160 ms in the experiments). In the paired condition, cue and reward were presented in the 10^th^ and 60^th^ time bins respectively. In the random condition, the cue was always presented in the 10^th^ time bin; whether or not the reward was presented in a trial was determined by drawing a uniformly random bin number in the range [0, 300] and the reward was presented in the trial if the bin number was in the range [21, 100], otherwise no reward was presented in the trial. A session consisted of 25 trials. In the DA-blockade condition, reward prediction was not updated during sessions 1–7 (in which the DA receptor antagonist was administered). For ease of comparison with the experimental data, the response probabilities were scaled to be between [−1, 1] in figures 3A and 4A. We reported each result, taking the mean of 20 simulations.

#### Statistical tests

We analyzed the effect of session on probability of responses for each group and condition. The difference in probability of sign-tracking responses vs. goal-tracking responses was examined using one-sample t-tests to determine if any preference is exhibited in each of the paired and the random conditions. Responses in the 8^th^ session were compared between groups of DA-blockade condition using an unpaired t-test for each group separately. Furthermore, responses in the 8^th^ session of the DA-blockade models were compared to that of the paired models responses in the 8^th^ session (using an unpaired t-test). We used a mixed model ANOVA with session as the repeated measure and stimulus (cue and reward) or group (sign- and goal-tracking) as the between-group measure to compare TD errors across paired and random conditions.

### SR task

In this two-choice task, animals could freely choose to nose-poke one of two ports at any time. An illuminated-lever (the experienced cue in the original PCA task) was presented for a short time interval (2 s) if the port designated as active by the experimenter was poked; otherwise (poking the inactive port) the cue was not presented. In this task, food rewards were not given. The relative bias in choosing between the active and inactive ports is thus considered to indicate the degree of attractiveness of the cue, i.e., how much presentation of the cue (due to its previous association with reward) would induce animals to choose the active port relative to the inactive port.

#### Simulation procedure

We simulated the task using revised action-dependent reward prediction learning model. For simplicity, we simulated the task using two-choice episodic trials with only one choice allowed in a trial. Each trial was divided into 3 time periods – port selection, outcome (lever or no-lever) and end-of-trial – with action selection allowed only in port selection period. This simplification of the SR task was to focus our simulation on measuring the relative preference of active ports in paired vs. random condition. In the experiments, animals performed PCA task in either paired or random condition before they were presented with the SR task. Accordingly, we simulated the SR task after the model learned the reward predictions in either paired or random condition.

**Figure 5:**
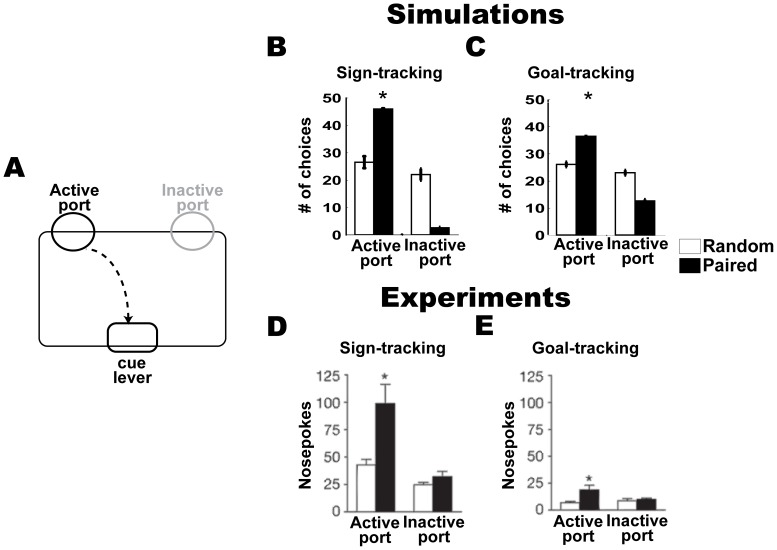
Secondary reinforcer task. (A) Task apparatus. In this task, a nose poke into the active or inactive port led to insertion of the cue into the chamber for 2 s or nothing occurred, respectively. The active and inactive ports were randomly pre-assigned for each animal. (B, C) Simulation results: number of choices, mean + s.e.m, of the active and inactive ports in sign-tracking (B) and goal-tracking (C) models with either paired (black bars) or random (white bars) cue-reward presentations in the PCA task. (D, E) Results of experiments [25]: number of nose pokes into the active and inactive ports in sign-tracking (D) and goal-tracking (E) rats that received either paired (black bars) or random (white bars) cue-reward presentations in the PCA task. Significantly different responses to active port in paired vs. random condition (P<0.01; t-test) are indicated with ‘*’.

Action-dependent reward predictions for the active (*Q_active_*_–*port*_) and inactive (*Q_inactive_*_–*port*_) ports were used to compute probability of choosing active or inactive port given by, *P_active_*_–*port*_ = *σ*(*β*(*Q_active_*_–*port*_ – *Q_inactive_*_–*port*_)). The action-dependent reward predictions were learned based on the reward prediction assigned to the outcome (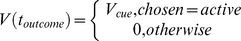). When the active-port was chosen, the cue was presented in the trial and at the end of the trial, the neural correlate of cue-evoked reward (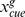) were updated. We ran 20 simulations for each condition (paired or random) each of 1000 trials and used the mean number of choices during port selection period. In Figure 5B and 5C, the observed number of choices was rescaled to be within the range [0, 50] (where the maximum value is the total number of trials divided by 20).

#### Statistical tests

The responses in the port selection period were analyzed using a 3-way ANOVA with the group (sign- vs. goal-tracking), condition (paired vs. random) and port (active vs. inactive) as independent variables and number of choices as dependent variable. The effect of condition or port on the group and effect of group or condition on port were analyzed using ANOVA.

## Results

We first examined the behavior of the models in the PCA task (Figure 3). The simulations confirmed our hypothesis on preference for sign- and goal-tracking behaviors due to differential learning of sign and goal components. Sign-tracking behavior gradually developed over sessions in models with relatively larger learning rates for reward prediction via TD error (*α* = 0.2) and smaller rates for correlates of cue-evoked reward presentation (*η* = 0.01) (Figure 3A; white-circles; sessions 1–12, P<0.001; one sample t-test). In contrast, goal-tracking behavior developed over sessions in models with larger and smaller rates of learning for cue-evoked reward representation correlates (*η* = 0.2) and TD error learning (*α* = 0.01) respectively (Figure 3A; black-diamonds; sessions 1–12, P<0.001; one sample t-test). When the cue and reward were unpaired (random condition: Methods), preference for neither sign- nor goal-tracking was observed (Figure 3A; black-circles and white-diamonds respectively). Response probability in both these models grew at the same rate and similar intensity, as found in the sign- and goal-tracking rats in the experiment (Figure 3B).

Interestingly, an intermediate group of rats were previously described [14] in the PCA task – initially these rats show preference for goal-tracking responses but after sufficient learning changed preference to sign-tracking responses. Although, this preference for sign-tracking responses remains lower than that of the sign-tracking group of rats. We tested if the model could explain these types of Pavlovian behaviors observed in the PCA task. We used learning rates (*α*, *η*) = (0.1, 0.1) that are intermediate to learning rates used in both sign- and goal-tracking groups and found the model showed preference for goal-tracking in early sessions and gradually developed preference for sign-tracking responses (Figure 4A; gray circles). The simulated intermediate learning rates model showed qualitatively similar responses over sessions as intermediate group of rats in the experiments (Figure 4B; gray circles). The model produces different response types in PCA task in different parameter regimes and thus can be used to model and understand the neural correlates of reward prediction in the sign- and goal-tracking rats.

We next measured the behavior of the models in the SR task (Figure 5A) and found that the cue was a significantly better secondary reinforcer in the sign-tracking group (Figure 5B) than in the goal-tracking group (Figure 5C). The number of responses toward the active-port was significantly greater in models that learned reward prediction in the paired condition than in models that learned reward prediction in the random condition in both the sign- and goal-tracking groups (i.e. significant effect of condition in both groups). But, the differences in the number of responses towards the active-port between the paired and random conditions were greater in the sign-tracking than in the goal-tracking group (group x condition interaction; F(1, 80) = 77, P<0.0001). These responses are consistent with those observed in the animal experiments, in which the rats were divided into sign-tracking (Figure 5D) and goal-tracking (Figure 5E) groups based on their behavior.

**Figure 6:**
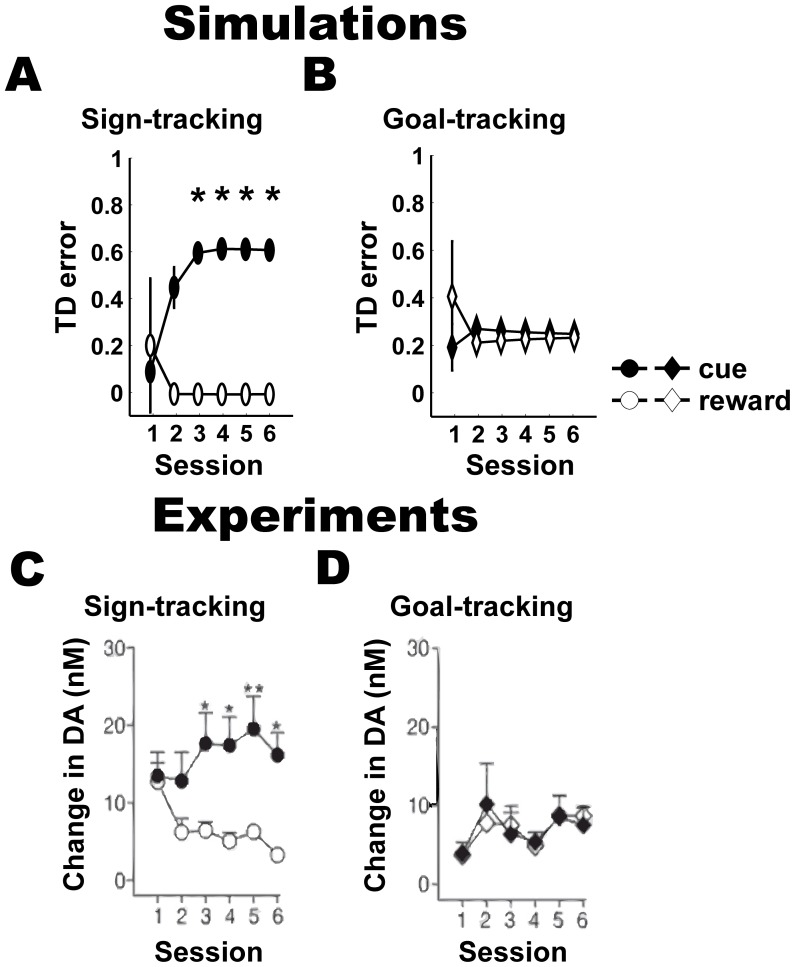
Temporal difference (TD) errors and phasic dopamine (DA) responses in the PCA task. (A, B) Simulations: temporal difference errors (a putative indicator of phasic DA activity), mean + s.e.m, at the time of cue and reward (white) in the sign-tracking (A) and goal-tracking (B) models. (C, D) Experiments [25]: peak DA concentration recorded in the core of the nucleus accumbens (mean + s.e.m), using fast-scan cyclic voltammetry and measured as change in peak DA concentration during the 5 s after cue or reward presentation averaged over 25 trials in each session, of sign-tracking (D) and goal-tracking (D) rats. Significantly different responses (P<0.01) between the cue and the reward are shown with ‘*’ (paired t-test).

We then evaluated the TD errors (Equation 2) of sign- and goal-tracking groups in the PCA task with DA activity in corresponding rats. First, we found that the TD errors in the sign-tracking group (Figure 6A) behaved qualitatively similarly to the DA activity in sign-tracking rats (Figure 6C). Specifically, we found that the sign-tracking group in paired condition exhibited TD errors that increased at the time of cue and decreased at the time of reward (Figure 6A; condition x session interaction; F(5, 40) = 12, P<0.005; compared to random condition). Second, we also found a correspondence between the TD error of the goal-tracking group (Figure 6B) and DA activity in goal-tracking rats (Figure 6B). The goal-tracking group in paired condition had persistent and similar TD errors at the time of cue and reward throughout the simulation (Figure 6B; condition x session interaction; F(5, 40) = 0.2, P = 0.3; compared to random condition). These TD errors are qualitatively consistent with the DA activity at the time of cue and reward presentation in goal-tracking rats (Figure 6D).

**Figure 7:**
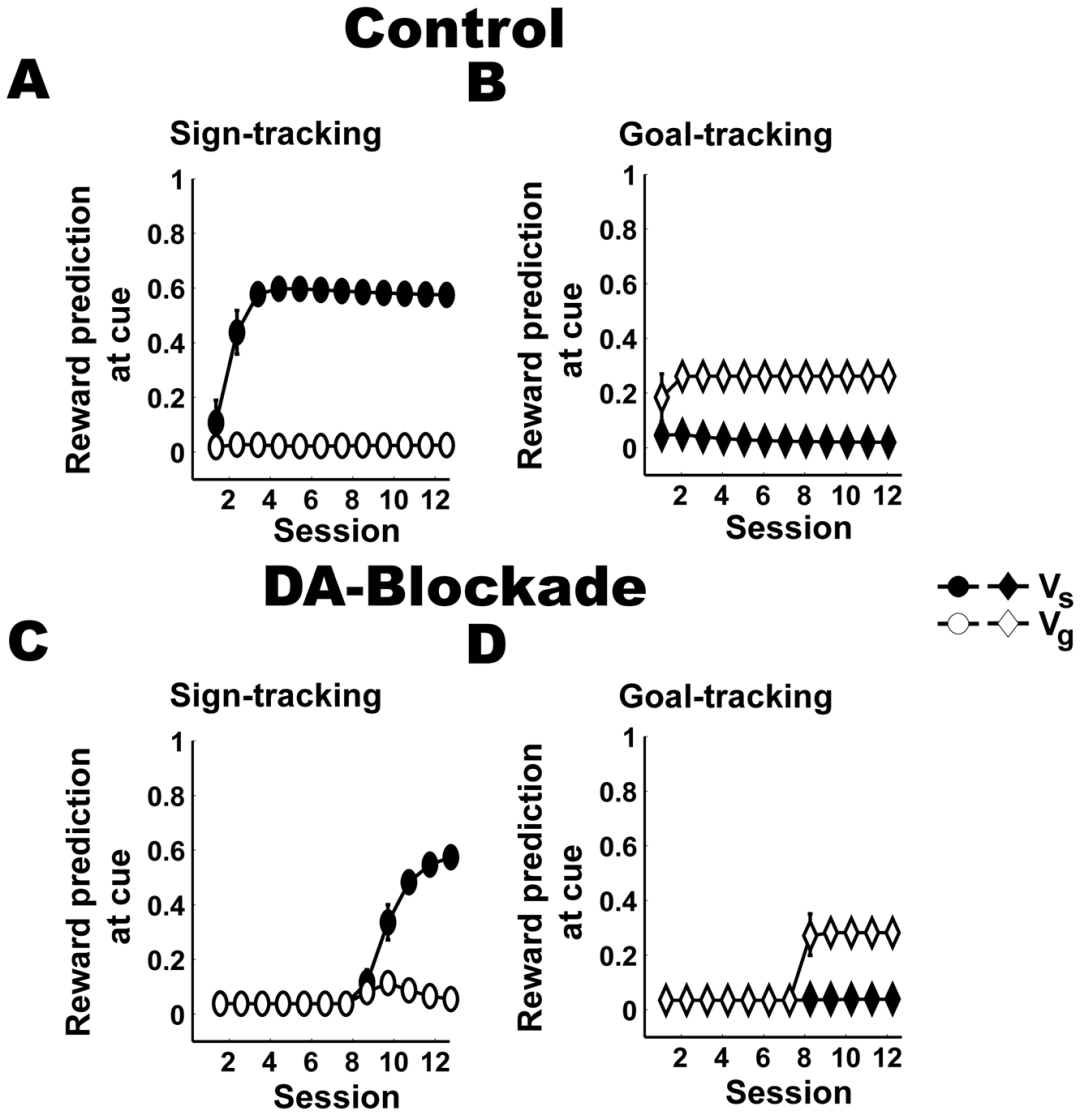
Components of reward prediction at the time of cue presentation in ST and GT. Reward predictions (*V*(*t*)) are learned as the sum of the sign (*V_s_*(*t*)) and the goal component (*V_g_*(*t*)), which are based on correlates of cue (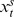) and cue-evoked reward (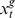) respectively. Reward prediction components evoked by the cue presentation are shown for both ST and GT groups across different sessions of the PCA task in paired (or control) (A, B) and DA-blockade (C, D) conditions.

We looked at the sign and goal reward prediction components at the time of cue presentation in both sign- and goal-tracking groups (Figure 7A, B). In the sign-tracking group, the sign component (Figure 7A; black circles) develops to a high value over sessions while the goal component (Figure 7A; white circles) remains at a very small value. The sign component thus becomes predominantly responsible for reward prediction in this group. In the goal-tracking group, the goal component (Figure 7B; white diamonds) increases to relatively larger value and sign component (Figure 7B; black diamonds), though small initially, slowly decreases over sessions. We should also notice that although the goal component in goal-tracking group (Figure 7B; white diamonds) increased it asymptotes at a lower level than the sign component in the sign-tracking group (Figure 7A; black circles). The increase in goal component and decrease in sign component leads to a larger role of goal-component in the goal-tracking group. Based on this data, we consider that the sign and goal component primarily determine the observed TD errors in sign- and goal-tracking groups respectively, i.e. larger TD error at the cue and smaller TD error at the reward in sign-tracking group compared to goal-tracking group. In addition, the fact that the sign component is larger in sign-tracking than in the goal-tracking group underlies the higher number of active-port choices in the sign-tracking group than in goal-tracking group in the SR task.

**Figure 8:**
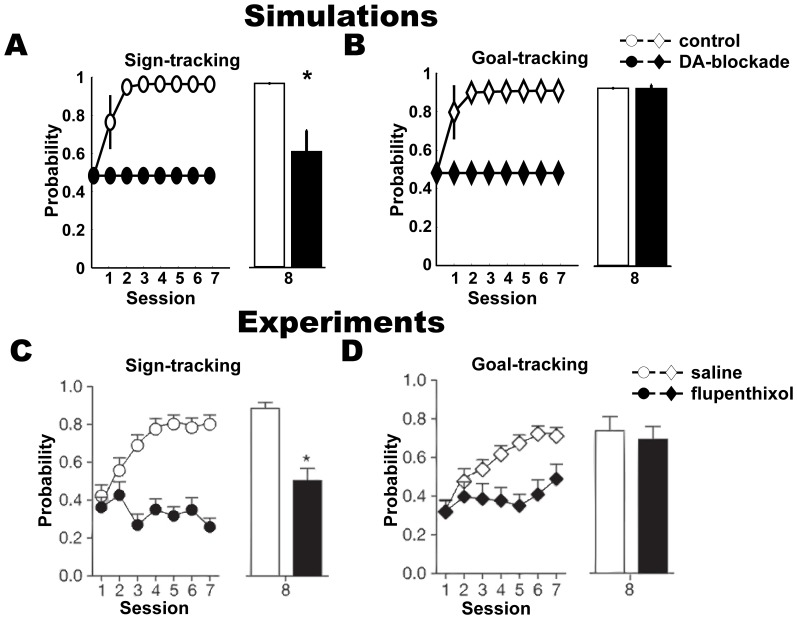
Recovery of TD error and DA led to rapid emergence of goal-tracking, but not of sign-tracking. **(A, B)** Simulations: (*Left*) probability of sign-tracking, mean + s.e.m, (A) and goal-tracking, mean + s.e.m, (B) in the models when reward prediction learning was present (white) or absent (black) in the first 7 sessions and (*Right*) corresponding probabilities in the 8^th^ session, mean + s.e.m, where reward prediction learning was present. (C, D) Experiments [25]: (*Left*) Probability of approaching the CS-lever, mean + s.e.m. in presumed sign-tracking rats (C) and food-tray, mean + s.e.m, in presumed goal-tracking rats (D) upon injection of flupenthixol (black) and saline (white) in the first 7 sessions and (*Right*) corresponding probabilities, mean + s.e.m, in the 8^th^ session where flupenthixol was not administered. Significantly different probabilities in 8^th^ session are indicated by ‘*’ (unpaired t-test).

We further investigated the role of TD errors in the learning of these responses in the PCA task simulations, in relation to the experimental findings with DA-receptor antagonist administered to the rats. Neither the sign- nor the goal-tracking group expressed corresponding responses during the sessions in which reward prediction was not updated (i.e., first 7 sessions with the presence of the DA-receptor antagonist in the rats; see Methods). During this phase, there is no reward prediction (Figure 7C and D; black circles and diamonds) and only the correlate of cue-evoked reward (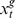) is updated based on associative learning (Equation 5). Since the goal-tracking group has higher rate of learning the correlate of cue-evoked reward (*η*) than the sign-tracking group, they will have higher 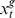 at the end of this phase (not shown). Due to the larger 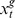 in goal-tracking group, when the reward prediction learning was restored in the 8^th^ session, the change in goal component (Equation 6) as well as change in reward prediction due to TD error (Equation 4) is larger in goal-tracking group than in sign-tracking group (Figure 7D; black diamonds and Figure 7C; black circles respectively). Thus, the goal-tracking group recovered goal-tracking responses in the 8^th^ session as soon as the reward prediction learning was restored (Figure 8B; P>0.1; unpaired t-test, 8^th^ session DA-blockade vs. 8^th^ session paired condition). In contrast, in the sign-tracking group, the magnitude of 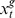 in the 8^th^ session of DA-blockade condition is similar to that of 1^st^ session of paired condition. Thus, no such immediate recovery was evident in the sign-tracking group (Figure 8A; P<0.001; unpaired t-test, 8^th^ session DA-blockade vs. 8^th^ session paired condition). These results correspond with observed behaviors in the experiments (Figure 8C, D).

## Discussion

This study introduced a new TD model that demonstrated a number of characteristics in correspondence with the experimental findings of sign- and goal-tracking: the respective behavioral responses and DA activity (putative TD error) in the PCA task, including the effect of DA antagonist on learning, and secondary reinforcer effect in the SR task. The TD model has two correlates that support reward prediction. One is the standard input in the RL description, i.e. the correlate of cue itself, but the other is the correlate of cue-evoked reward learned via cue-reward association. The reward prediction, and its components, is learned using TD error. The learning of reward prediction is also affected by learning through the correlates of cue-evoked reward, which is independent of TD learning. We hypothesized that the relative prediction due to the standard and new correlates leads to sign- or goal-tracking response; differential rates of learning in leads to variability in the behavioral response. We examined these effects by simulating our models using two different set of learning rates: *α* and *η*, for learning reward prediction (via TD error) and the correlates of cue-evoked reward representation.

There is a different view on the discrepancy between the findings on the sign- and goal-tracking and the standard RL account based on the distinction between model-free and model-based RL [27]–[29]. In brief, model-free RL is associated with learning via DA activity, whereas the model-based RL involves additional learning about the environment, or the task (e.g., the location of food-tray). In this view, sign- and goal-tracking rats are differentiated based on relative influence of model-free and model-based RL, respectively. We assumed the neural correlates relating to cue-reward association are learned, similar to this view, but the goal-tracking response in our model is learning in the model-free RL, i.e. corresponding prediction is learned using DA activity. These two accounts can be distinguished using experimental manipulations probing the difference between model-based and model-free RL mechanisms, such as devaluation of rewards to verify the role of model-based RL in goal-tracking rats. Our proposal is related to other works in a broader context as well [30], [31], on preparatory vs. consummatory responses [20], stimulus-substitution vs. stimulus-response conditioning theories [11], [14], and Pavlovian-instrumental transfer [32], [33]. Intriguingly, these studies often point to different regions of the brain involved in cue- or reward specificity in reward predictions – especially the role of core vs. shell of the nucleus accumbens; other studies have shown that the cue-evoked neural correlate may change in orbitofrontal cortex, according to the motivation (reward specific) [34]–[37] but perhaps not so much in the insular cortex and amygdala (cue specific) [38], [39].

We used a number of simplifying assumptions in our modeling in this study with the aim of instantiating our proposal in a simple formulation. First, we did not include any mechanisms to arbitrate the contribution of two components in reward prediction. Others have used reward uncertainty to arbitrate between two competing reward prediction components [2], [40]–[42]. Second, more sophisticated forms of cue-reward association could be used for learning the correlates of cue-evoked reward. These improvements might help address dynamic aspects of the sign- and goal-tracking, e.g., the role of distance, timing, and contingency between the cue and reward [17], [43], [44]. In terms of computational techniques, the predictive state [45], [46] or successor [47], [48] representations may be applied. Third, it has been shown that sign-tracking rats are generally higher responding in a novel task compared to goal-tracking rats [14], [16]. We did not address this issue or response times in the behavior. Related to this, we used a simplified simulation of SR task with only choice allowed in the trial and mainly focused on the differences between groups in number of active-port choices. But, we also found that the preference for inactive port in paired condition was much lower in sign-tracking models than in sign-tracking rats. Finally, we followed the RL hypothesis that the firing rate of single DA neurons encodes TD errors. In contrast to model TD errors, the experimental measured DA activity is the peak concentration of dopamine in the core of the nucleus accumbens. It is unclear how the experimental measurement should be compared to TD errors and so we did not address the discrepancy between simulated TD errors and experimental measurements.

In Flagel et al 2011[25], the cue became “incentive” stimulus for sign-tracking rats, as it became not only predictive but also “attractive” (approach in PCA task) and “wanted” (preference for active-port in SR task). In our model, we did not explicitly define such a unified concept that is acquired by the stimulus. Instead the model assumed two components in reward prediction and associated with the two responses. In this view, the “incentive salience” of the stimulus is, not a unified property acquired by the stimulus, but a consequence of interactions between the two components during the learning of reward predictions. The differential role of cue-reward association learning on reward prediction also points to a role for DA activity independent learning in incentive salience attribution in sign- and goal-tracking rats.

In summary, this study proposed that DA activity might still encode reward prediction error signals, despite the discrepancy between the findings on sign- or goal-tracking rats. However, the influence of DA activity in learning reward predictions can vary between the individuals. We validated our proposal by simulating the models and comparing them to experimental results. We conceptualized the notion that the neural correlates that support reward prediction in the RL may have multiple origins, i.e. correlate of cue itself as well as cue-evoked representation of associated rewards. The results of this study demonstrate the importance of investigating the neural correlates underlying reward prediction [18], [19], [49], [50] in future studies of conditioning.
